# Taxonomic and Functional Metrics of Ciliates and Amoeboid Protists in Response to Stream Revitalization

**DOI:** 10.3389/fmicb.2022.842395

**Published:** 2022-04-01

**Authors:** Vesna Gulin, Barbara Vlaičević, Mirela Sertić Perić, Fran Rebrina, Renata Matoničkin Kepčija

**Affiliations:** ^1^Department of Biology, Faculty of Science, University of Zagreb, Zagreb, Croatia; ^2^Department of Biology, Josip Juraj Strossmayer University of Osijek, Osijek, Croatia

**Keywords:** eukaryotic, single-celled, freshwater, soil, phagotrophic, functional traits, protozoa

## Abstract

Tufa-depositing streams provide great microhabitat complexity and are therefore inhabited by various periphytic phagotrophic organisms such as ciliates and amoeboid protists. Recent removal of invasive plant species *Ailanthus altissima* (Mill.) Swinge from the Skradinski buk tufa barrier (Krka National Park, Croatia) resulted in changes in the barrier hydromorphology including the reactivation (revitalization) of dry streams. The objective of this study was to investigate: (1) the taxonomic and functional response of periphytic ciliates and amoeboid protists to stream revitalization by comparing taxonomic (i.e., abundance, species richness and diversity) and functional (i.e., functional diversity) metrics between revitalized (N) and control sites (C) during 1 and 2-months immersion period; (2) which environmental and (3) periphyton-associated factors shape the taxonomic and functional metrics and to what extent; (4) how duration of immersion affects taxonomic and functional metrics at revitalized sites. Our results showed that taxonomic and functional metrics of ciliates and amoeboid protists responded to the prevailing conditions characteristic of revitalized tufa-depositing streams: changing hydrology (occasional high flow or drought), soil drainage, and extensive inorganic matter, i.e., tufa deposition, although their responses were somewhat different. The two assemblages also showed different responses of taxonomic and functional metrics with respect to immersion duration: while the taxonomic and functional diversity of ciliates at N sites increased with longer immersion, indicating niche diversification, those of amoeboid protists hardly changed with time. Our results suggest that a comprehensive analysis of taxonomic and functional metrics of ciliates and amoeboid protists could be a good proxy for assessing revitalization of tufa-depositing streams. However, the temporal component should always be considered when conducting such studies, as the colonization processes of ciliates and amoeboid protists are quite complex, especially in tufa-depositing streams.

## Introduction

Protists are the most diverse and widespread eukaryotes (de Vargas et al., 2015; Adl et al., 2019; Sieber et al., 2020). They perform important ecosystem functions and play a fundamental part in aquatic food webs (Weisse et al., 2016). Phagotrophic protists (those that utilize food through phagocytosis) have recently been shown to be even more diverse than their phototrophic counterparts (Singer et al., 2021). As the most important microbial predators in aquatic and soil ecosystems, phagotrophic protists transfer carbon and energy between micro- and macrofauna, significantly influencing ecosystem-level processes (Sherr and Sherr, 1984, 2002; Risse-Buhl et al., 2015; Geisen et al., 2017).

Due to their ubiquity, abundance and sensitivity, phagotrophic protists have been recognized as excellent bioindicators in a variety of environments (Payne, 2013). Ciliates (Ciliophora) and testate amoebae (Amoebozoa and Rhizaria) are very common groups of phagotrophic protists in freshwater and soil ecosystems (Singer et al., 2021). They are also the best-studied protists, partly because they are easy to isolate and have a distinctive morphology (Foissner, 1999; Todorov and Bankov, 2019). Naked amoebae, on the other hand, are understudied due to their smaller size and a lack of knowledge on their morphological features, although they dominate many protist communities, particularly in periphyton and benthos, as well as in soil (Coleman and Wall, 2015; Geisen, 2016; Singer et al., 2021).

Different protist groups may play opposing or complementary roles in ecosystem functioning. Thus, analyzing their functional diversity (FD) may enhance our understanding of various protist traits and their role in the ecosystem (Dumack et al., 2020). Functional diversity analysis is based on linking ecosystem processes to species’ functional traits, i.e., organisms’ characteristics that influence not only their fitness, but also their interactions with the environment (Nock et al., 2016). Trait-based approaches offer an opportunity to understand the relationship between microbial diversity and ecosystem functioning better than taxonomy-based approaches, especially since taxonomic and functional diversity are not always linearly correlated (Laliberté and Legendre, 2010). However, the trait-based approach has rarely been applied to protists (Weisse, 2017; Fiore-Donno et al., 2019).

Phagotrophic protists have already been recognized as good bioindicators of the karst environments’ (e.g., tufa-depositing lakes, rivers, and streams) quality (Primc-Habdija et al., 2001; Matoničkin Kepčija et al., 2011; Kulaš et al., 2021). A recent study by Gulin et al. (2021) showed that periphyton responds to revitalization of dry tufa-depositing streams, opening new possibilities for the application of periphytic organisms as proxies of the stream revitalization success. The study focused on evaluating the response of periphyton based on sampling natural substrates, i.e., stream substrate containing tufa particles, which allowed analysis not only of the abundance and diversity of periphytic taxa, but also of the granulometric and mineral constituents of the substrate itself. However, the method of sampling with a corer proved too aggressive for periphytic organisms, resulting in low abundance and diversity of periphyton. In contrast, the present study is an improvement, because it is based on evaluating the response by monitoring periphyton development on artificial substrates over time so that the periphyton can be studied directly without damaging it. It focuses on phagotrophic protists (ciliates and amoeboid protists) and analyses both their taxonomic and functional diversity. Since periphyton exhibits complex spatio-temporal colonization trends (Franco et al., 1998; Risse-Buhl and Küsel, 2009), 1 and 2-months immersion periods were covered.

Our first objective was to assess whether taxonomic (abundance, species richness and diversity) and functional (i.e., functional diversity) metrics of ciliates and amoeboid protists differed between control (C) sites (located within permanent streams) and revitalized (N) sites (located within reactivated streams). We expected higher abundance and diversity (taxonomic and functional) of ciliates and amoeboid protists at revitalized sites than at control sites. This was based on the fact that conditions that prevail at reactivated streams due to newly created waterways and soil drainage, represent a positive disturbance, which can improve the diversity of habitats, species and their survival strategies (Timoner et al., 2014). A better understanding of the effects of disturbance on functional diversity is critical because functional diversity is likely related to ecosystem resilience, i.e., the capacity of a system to absorb shocks, reorganize, and maintain the same structure and function (Walker, 1995).

The second objective was to determine the influence of several environmental factors (flow velocity, temperature, dissolved oxygen concentration, pH, conductivity, alkalinity, total water hardness, concentrations of nitrites, nitrates and orthophosphates, and chemical oxygen demand) on the taxonomic and functional metrics of ciliates and amoeboid protists. Some of these factors have already shown a significant effect on the assemblage metrics of periphytic taxa in revitalized tufa streams (Gulin et al., 2021). Based on previous findings, we expected several organic matter associated factors (e.g., chemical oxygen demand, orthophosphates) to increase the abundance and taxonomic diversity of phagotrophic protists while others (e.g., nitrites) were expected to yield the opposite (decreasing) effect (Gulin et al., 2021).

Our third objective was to estimate how periphyton-associated factors (organic and inorganic matter (tufa) content, chlorophyll *a* concentration) affect the taxonomic and functional metrics of ciliates and amoeboid protists. Based on the findings of Gulin et al. (2021), we expected that increased organic matter content (and subsequently chlorophyll *a* concentration) would lead to increased abundance of protists. On the other hand, we expected that increased inorganic matter (i.e., tufa) content would decrease the taxonomic and functional diversity of protists as intense tufa deposition can lead to detachment and sloughing (Pitois et al., 2001, 2003).

Finally, our fourth objective was to examine the extent to which duration of immersion in water influences changes in taxonomic and functional metrics of ciliates and amoeboid protists at revitalized sites. We expected to observe a trend of increasing values of taxonomic and functional metrics with longer immersion duration, for both ciliates and amoeboid protists from revitalized sites.

The links between ecosystem functioning and the taxonomic and functional diversity of phagotrophic protists could be fundamental to resource management and conservation planning, particularly in protected and sensitive areas such as national parks. The results of this research would make an important contribution to understanding fragile and complex tufa-depositing ecosystems.

## Materials and Methods

### Study Area

**FIGURE 1:**
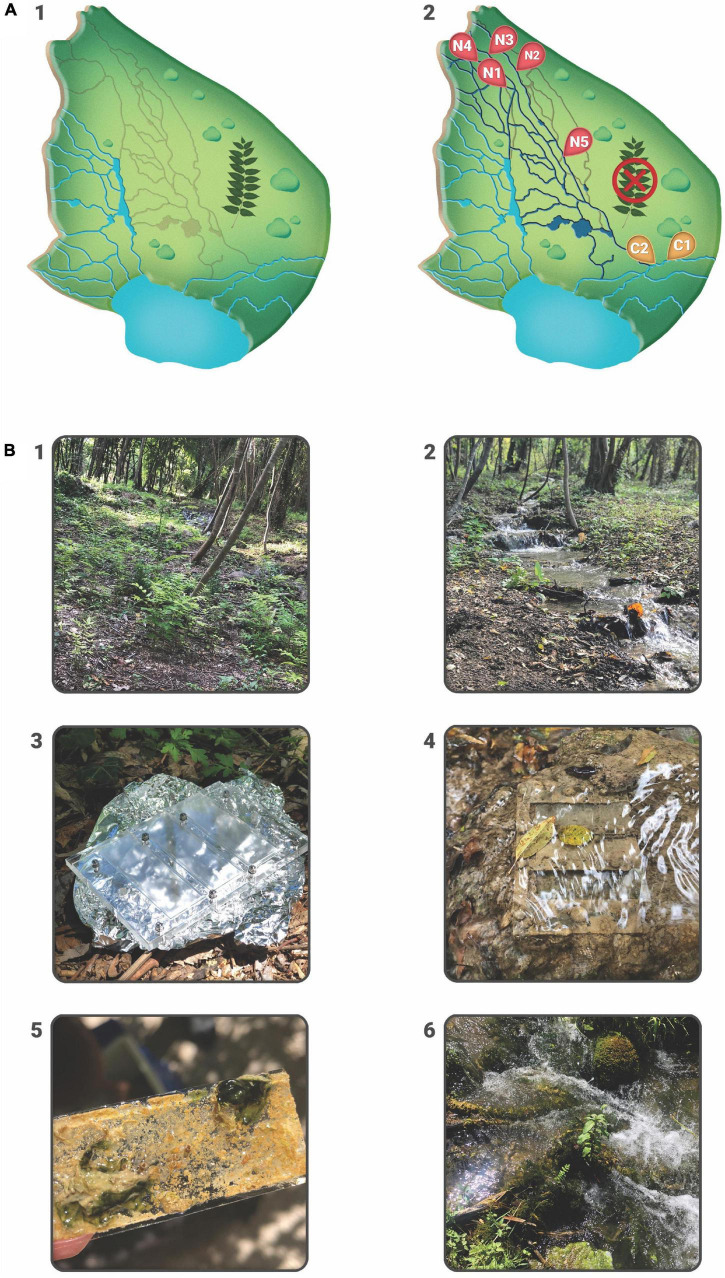
**(A)** Experimental plot at Skradinski buk barrier with graphical presentation before and after the removal of *A. altissima*. Gray lines in A1 represent dry streams which were revitalized upon invasive plant removal. A2 shows seven sampling sites: two control sites located within permanent streams, C1 and C2, and five revitalized sites N1–N5. **(B)** Details from the experimental plot: 1—dry streams overgrown with *A. altissima*, 2—revitalized streams upon the removal of *A. altissima*, 3—3D printed Plexiglass carriers, 4—Plexiglass carriers with glass slides placed in stream bed, 5—a detail of a glass slide from N site with deposited inorganic matter, i.e., tufa, 6—well-developed moss cover from C1 site.

The study was conducted on a 1 ha experimental plot at the Skradinski buk tufa barrier-the longest and final tufa barrier in the watercourse of the Krka River, a karst river in the Dinaric region of Croatia, protected as a national park since 1985. Following an extensive aerial survey (Phantom 4) and detailed vegetation mapping, which determined that the invasive plant *Ailanthus altissima* (Mill.) Swinge (tree of heaven) dominated the area and was causing the dryness of the barrier due to its strong root system, individual specimens of the invasive tree were mechanically removed in August 2017 with permission from the Croatian Agency for Environment and Nature. Within 2 months of the removal, five streams in the experimental plot, that had previously dried up completely, were reactivated. The sampling design included seven sampling sites selected after removal of an invasive plant species: two control sites (C) in permanent streams where water was present before and after plant removal, C1 (15.966381, 43.805752)-control site where water had been present before and after the removal of *A. altissima* and displaying well-developed moss cover; C2 (15.966279, 43.805772)-control site without moss cover and five revitalized (N) sites: N1 (15.965449, 43.806438), N2 (15.965324, 43.806624), N3 (15.965246, 43.806541), N4 (15.965186, 43.806489), N5 (15.965538, 43.806225), representing newly reactivated streams (Figure 1).

### Sampling Design and Identification

**FIGURE 2:**
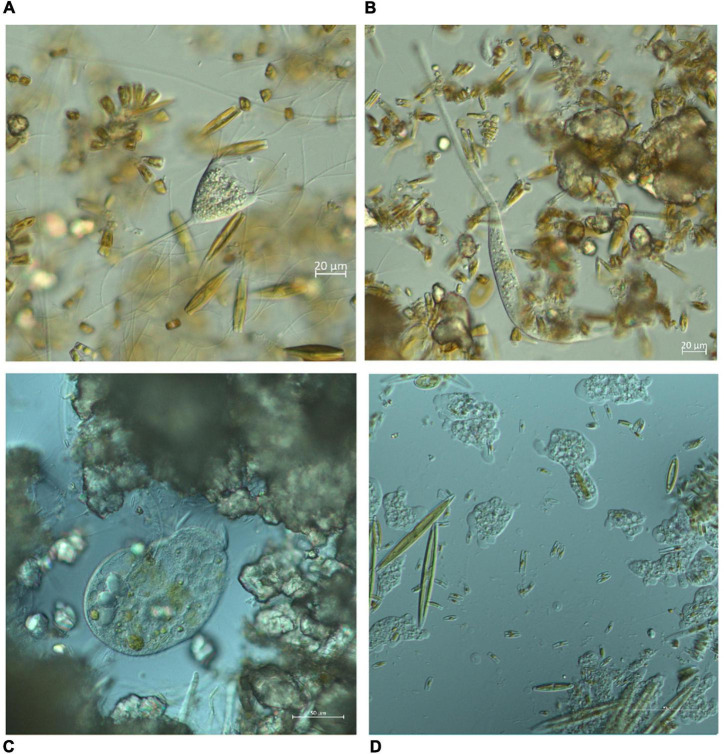
Photomicrographs of different organisms observed during the study period. The organisms were examined directly under the microscope using Zeiss Axioimager A2 with DIC objectives and captured using Axiocam 305 digital camera. **(A)** Ciliate *Acineta* sp., **(B)** ciliate *Lacrymaria* sp., **(C)** ciliate *Stentor* sp., **(D)** various naked amoebas.

On May 10, 2019, 14 3D-printed Plexiglass carriers were placed, two per each sampling site, each containing three glass slides (7.6 × 2.6 cm) to serve as artificial substrates for periphyton development (Figure 1). Prior to immersion into the stream, the slides were washed in detergent, 1 M hydrochloric acid and distilled water. The total effective surface area of each glass slide was 17.18 cm^2^ because it was partially covered by Plexiglass. Sampling was designed to immerse slides in the water for a period of 1 or 2 months, with three slides collected per sampling event at each site. Due to the high seasonality of the Krka River (Schöll et al., 2012), four sampling events were chosen for each immersion period, covering four different seasons. The dates on which slides were placed in the water and collected for the 1-month period were May 10–June 10, 2019 (spring), June 10–July 15, 2019 (summer), September 30–November 3, 2019 (autumn), February 2–March 8, 2020 (winter). The dates on which the slides were immersed for the 2-months period were placed and collected were June 10–August 15, 2019 (summer), September 30–December 2, 2019 (autumn), December 2, 2019–February 2, 2020 (winter), March 8–June 5, 2020 (spring, immersion period prolonged due to COVID-19 pandemic). A total of 168 slides were placed, 84 for the 1-month immersion period and another 84 for the 2-months immersion period. However, due to unpredictable events such as disturbance by visitors (sampling sites were located in close proximity to visitor trails and probably aroused curiosity) and seasonal drying of streams, only 55 slides were collected for the 1-month immersion period and 52 for the 2-months immersion period. More slides were collected from N sites due to the higher number of sampling sites in the first place. After collection, the slides were transferred to plastic containers filled with a small amount of ambient water and stored at 4°C in the dark. They were then examined directly under the microscope using Zeiss Axioimager A2 with DIC objectives and Axiocam 305 digital camera, within 24 h of sampling, except for few slides that were examined within 48 h. Ciliates and amoeboid protists were identified at species level using Zen 2.4 imaging software and relevant literature (Kahl, 1930; Page, 1976, 1991; Foissner et al., 1991, 1992, 1994, 1995; Foissner and Berger, 1996; Smirnov and Brown, 2004; Smirnov et al., 2011; Todorov and Bankov, 2019). For each taxon, 10–15 photomicrographs were taken and subjected to morphometric measurement (Figure 2). Additional video clips were used to record movements and distinguishing features.

### Environmental Factors

The following environmental factors were measured at each sampling site using the respective portable field meters: temperature (T) and dissolved oxygen concentration (DO) (oximeter OXI 96, WTW GmbH, Weilheim, Germany), pH (pH meter 330i, WTW GmbH, Weilheim, Germany), conductivity (Cond) (conductometer Sension 5, Hach, Loveland, Colorado, United States), and flow velocity (FV) (flow velocity meter P600, Dostmann electronic GmbH, Wertheim- Reicholzheim, Germany). An additional 1 L water sample was collected and stored at 4°C for subsequent laboratory analysis of the water. This included the analysis of alkalinity (Alk), total water hardness (TWH), concentrations of nitrite (N-NO_2_^–^), nitrate (N-NO_3_^–^) and orthophosphate (P-PO_4_^3–^) (according to APHA, 1985), and total chemical oxygen demand (COD) using the standardized acidic potassium permanganate titrimetric method (Deutsches Institut für Normung, 1986).

### Periphyton-Associated Factors (Organic/Inorganic Matter Content and Chlorophyll *a* Concentration)

After microscopic examination, periphyton from each slide was divided into two equal parts-one was used to determine the organic/inorganic content and the other was scraped to measure the chlorophyll *a* concentration (a proxy of primary productivity) (Falkowski and Raven, 1997). For the determination of organic matter content as ash-free dry mass, the samples were dried at 104°C to constant weight, then ashed at 400°C for 4 h and reweighed. The mass difference between the dried sample and the ashed sample was used to express the amount of organic matter while the mass difference between the ashed sample and the glass slide was used to express the amount of inorganic matter, i.e., deposited tufa. Values were expressed as the mass of organic/inorganic matter (mg) content per cm^2^ surface area. Chlorophyll *a* concentration was determined by the ethanol extraction method (Nusch, 1980). Values were expressed as mass of chlorophyll *a* (μg) per 8.59 cm^2^ surface area.

### Statistical Analyses

#### Data Exploration and Visualization

The data were summarized and displayed using standard statistical measures (mean and standard deviation) and graphically presented to illustrate possible trends using ggplot2 package v. 3.3.5 (Wickham, 2016) in R v. 4.1.1 (R Core Team, 2021).

Species richness, Shannon and Simpson diversity indices were calculated independently for ciliates and amoeboid protists as a measure of alpha diversity using R vegan package v. 2.5.7 (Oksanen et al., 2020). Diversity indices were subsequently converted to the effective number of species (true diversity) following a procedure proposed by Jost (2006, 2007). Abundance was calculated independently for ciliates and amoeboid protists as the number of individuals per cm^2^ surface area for 1 and 2-months immersion periods.

Functional traits were assigned to each species examined in this study based on the relevant literature for ciliates and amoeboid protists (Foissner and Berger, 1996; Foissner et al., 2002; Adl et al., 2019; Fiore-Donno et al., 2019). Ciliates were classified into categories (functional groups) based on the following functional traits: food source, feeding strategy, ecosystem preference, habitat preference, motility, mode of locomotion and life form. Likewise, the amoeboid protists were assigned to the following functional categories (groups): food source, habitat, and morphology (the presence or absence of shell). A detailed overview of the criteria for classification can be found in Supplementary Tables 1, 2.

To minimize the effects of qualitative and quantitative selection of functional traits on the results of functional diversity measures that are shown to be potentially significant (Pakeman, 2014; Zihao et al., 2021), we focused on the robust measures of functional dispersion (FDis) and RaoQ quadratic diversity (RaoQ) to quantify the functional diversity of ciliates and amoeboid protists. FDis is defined as the weighted mean distance in multidimensional trait space of individual species to the centroid of all species, where the weights are the relative abundances of the species (Laliberté and Legendre, 2010; Cappelatti et al., 2020). RaoQ is defined as the sum of pairwise distances between species in multidimensional trait space weighted by their relative abundance (Ricotta and Moretti, 2011). By construction, the two functional dispersion indices are not influenced by species richness (Teittinen and Virta, 2021). FDis and RaoQ values were calculated in R using the FD package v. 1.0–12 (Laliberté and Legendre, 2010; Laliberté et al., 2014). Indices of functional diversity were calculated separately for ciliate and amoeboid protist assemblages at C and N sites for the 1 and 2-months immersion periods.

#### Data Analysis

Each of the four data sets (1 and 2-months immersion periods for ciliates and amoeboid protists), environmental data and data regarding periphyton-associated factors were tested for normality using Shapiro-Wilk’s test in R prior to further analysis. Since the data were not normally distributed (Shapiro-Wilk’s test, *p* < 0.05) and sphericity was violated (Mauchly’s test, *p* < 0.05), differences in environmental and periphyton-associated factors between C and N sites were tested using the analysis of similarity (ANOSIM), a non-parametric test for evaluating a dissimilarity matrix instead of raw data (Clarke and Warwick, 1994) from the R package vegan v. 2.5.7 (Oksanen et al., 2020).

Generalized linear mixed models (GLMMs) were constructed using SPSS Statistics ver. 28.0 (IBM Corp, 2021) to test for differences in ciliate and amoeboid protist assemblages between C and N sites for the 1 and 2-months immersion periods. The taxonomic metrics considered were: abundance, species richness, taxonomic (True) diversity derived from the Shannon and Simpson indices while the functional diversity indices included FDis and RaoQ. Only those environmental factors that showed significant differences (ANOSIM) between C and N sites were selected as inputs to the models. GLMMs with the variables “site,” “COD,” “TWH” and “nitrites” as fixed effects were constructed to evaluate the relationships between 1-month assemblages of ciliates and amoeboid protists and environmental factors. For the 2-months immersion period, the variables “site,” “DO,” “conductivity,” “pH,” “COD,” “alkalinity,” “TWH” and “orthophosphates” were selected as fixed effects. The variables “replicate” and “season” were included as random effects for both model variations, by recommendation of Jost (2007).

Additional GLMMs with periphyton-associated factors (organic and inorganic matter content, chlorophyll *a* concentration) as fixed effects were constructed for both ciliate and amoeboid protist assemblages for the 1 and 2-months immersion periods. Due to repeated measures sampling setup, first-order autoregressive (AR1) covariance structure of random effects was assumed in all models constructed within this study (Field, 2009).

## Results

### Environmental Factors

**FIGURE 3:**
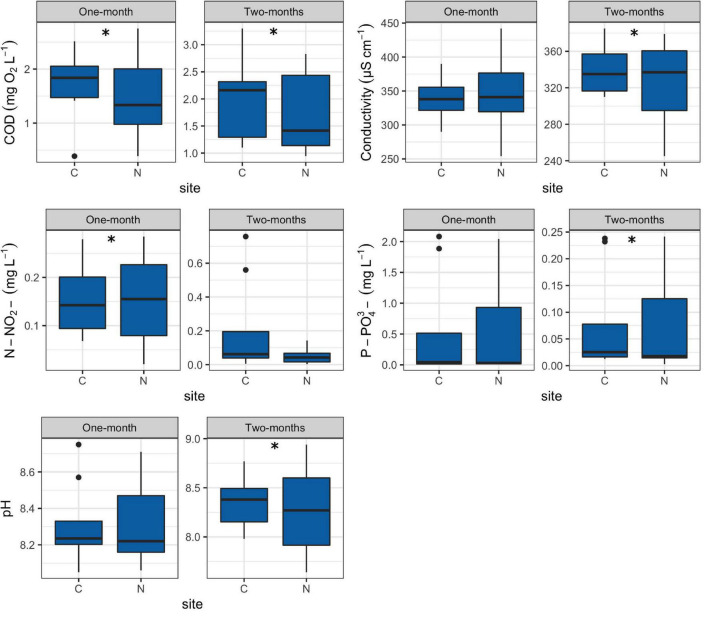
Box plots showing selected environmental factors at control (C) and revitalized sites (N) for the 1 and 2-months immersion periods. Asterisk symbol (*) indicates statistically significant differences among estimated means (ANOSIM, *p* < 0.05). Upper and lower edges of the boxes are the first and third quartiles; the line inside the box represents the median; individual dots are outliers. Abbreviations: COD—total chemical oxygen demand, N-NO_2_^–^—nitrites, P-PO_4_^3–^—orthophosphates.

Values of all environmental factors measured in this research can be found in Supplementary Table 3. The COD values for the 1-month period at N sites were significantly lower (*R* = 0.45, *p* = 0.015) in comparison to C sites while the opposite was found for nitrites (*R* = 0.64, *p* = 0.007) (Figure 3).

For the 2-months immersion period, N sites had significantly lower values of COD (*R* = 0.52, *p* = 0.004), pH (*R* = 0.69, *p* = 0.015) and orthophosphates (*R* = 0.70, *p* = 0.001), while higher values of conductivity (*R* = 0.91, *p* = 0.004) in comparison to C sites (Figure 3).

### Periphyton-Associated Factors

**FIGURE 4:**
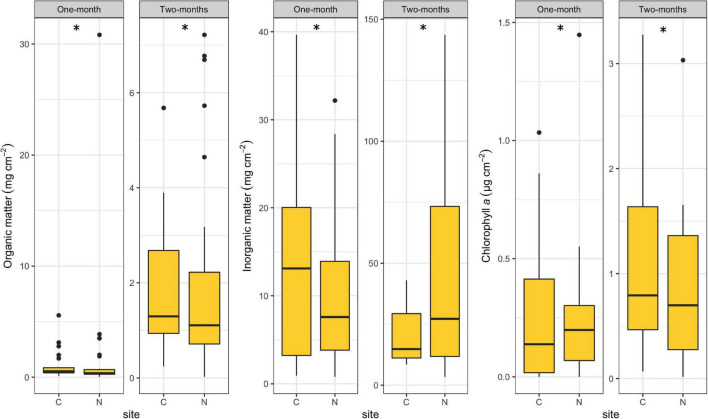
Box plots showing periphyton-associated factors at control (C) and revitalized sites (N) for the 1 and 2-months immersion periods. Asterisk symbol (*) indicates statistically significant differences among estimated means (ANOSIM, *p* < 0.05). Upper and lower edges of the boxes are the first and third quartiles; the line inside the box represents the median; individual dots are outliers.

All periphyton-associated factors differed significantly between C and N sites for both immersion periods (*R* = 1.00, *p* < 0.001). Organic matter content was lower at N sites for both periods (Figure 4), but it could be noticed that N sites occasionally had extremely high values (30.83 mg per cm^2^ during the 1-month period and 7.22 mg per cm^2^ during the 2-months period), while C sites had no such extreme outliers. Inorganic matter content was lower at N sites than at the C sites during the 1-month period, but showed the opposite during the 2-months period, reaching record values of 143.60 mg per cm^2^. Chlorophyll *a* concentration at N sites was higher compared to C sites during the 1-month period and lower during the 2-months period. However, it could be noticed that N sites, unlike C sites, had some extremely high chlorophyll *a* concentrations during both periods.

### Taxonomic and Functional Metrics of Ciliate Assemblages

**FIGURE 5:**
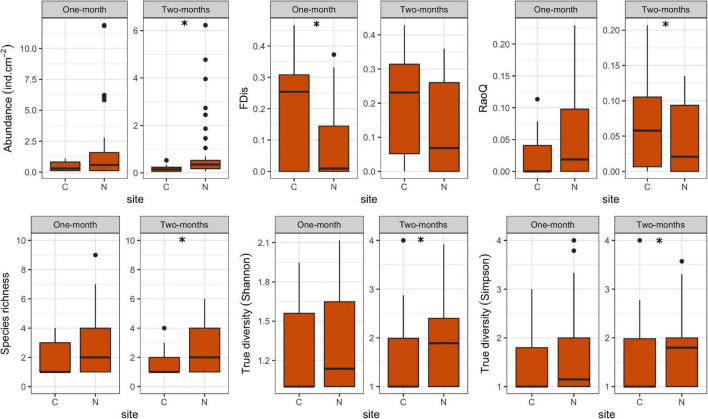
Box plots showing ciliate taxonomic and functional metrics at control (C) and revitalized sites (N) for the 1 and 2-months immersion periods. Asterisk symbol (*) indicates statistically significant differences among estimated means (GLMM, *p* < 0.05). Upper and lower edges of the boxes are the first and third quartiles; the line inside the box represents the median; individual dots are outliers.

In this study, a total of 78 ciliate species were identified, most of them (48) at N sites. Ciliate taxonomic metrics (abundance, species richness, Shannon and Simpson derived True diversity indices) were higher at N sites than at C sites for both immersion periods (Figure 5). Although no significant differences were confirmed by GLMMs with environmental and periphyton-associated factors for the 1-month period, all taxonomic metrics showed significant differences between N sites and C sites for the 2-months period (Tables 1, 2). As for correlations between taxonomic metrics and environmental factors, no significant correlations were found for the 1-month period, but several were observed for the 2-months period: abundance was significantly positively correlated with COD, species richness was significantly negatively correlated with conductivity, while True diversity values (Shannon and Simpson) were significantly positively correlated with pH and orthophosphates but significantly negatively correlated with conductivity (Table 3). Periphyton-associated factors were found to have significant correlations with taxonomic metrics for both 1 and 2-months periods (Table 4). All taxonomic metrics except abundance were positively correlated with inorganic matter and negatively correlated with organic matter and chlorophyll *a* for the 1-month period. The opposite was found for the 2-months period when all taxonomic metrics were positively correlated with organic matter and chlorophyll *a*, while negatively correlated with inorganic matter. Significant correlations were found for species richness and True diversity (Shannon) values for both periods and abundance for the 2-months period.

**TABLE 1:** GLMM (full model) output showing differences in ciliate assemblage metrics between control and revitalized sites (1 and 2-months immersion periods) with sites and environmental factors as fixed effects, and season and replicate as random effects.

	Assemblage parameter	*F*	*p*	d.f	d.f. corrected	p C-N
**One-month**	Abundance	1.092	0.301	1	50	0.282
	Species richness	0.982	0.327	1	50	0.309
	True diversity (Shannon)	0.064	0.801	1	50	0.801
	True diversity (Simpson)	0.194	0.661	1	50	0.657
	FDis	6.054	**0.017**	1	50	0.287
	RaoQ	0.186	0.668	1	50	0.655
**Two-months**	Abundance	10.583	**0.001**	1	43	**0.001**
	Species richness	5.314	**0.026**	1	43	**0.028**
	True diversity (Shannon)	5.988	**0.019**	1	43	**0.0014**
	True diversity (Simpson)	4.349	**0.043**	1	43	**0.035**
	FDis	0.261	0.612	1	43	0.754
	RaoQ	9.931	**0.003**	1	43	**0.035**

*Statistically significant effects (p < 0.05) are reported in bold. F, F statistic; d.f., degrees of freedom.*

**TABLE 2:** GLMM (full model) output showing differences in ciliate assemblage metrics between control and revitalized sites (1 and 2-months immersion periods) with sites and periphyton-associated factors as fixed effects, and season and replicate as random effects.

	Assemblage parameter	*F*	*p*	d.f	d.f. corrected	p C-N
**One-month**	Abundance	3.038	0.087	1	50	**0.046**
	Species richness	0.126	0.724	1	50	0.725
	True diversity (Shannon)	0.124	0.726	1	50	0.727
	True diversity (Simpson)	0.520	0.474	1	50	0.477
	FDis	26.586	**< 0.001**	1	50	**< 0.001**
	RaoQ	1.939	0.184	1	50	0.242
**Two-months**	Abundance	23.467	**< 0.001**	1	47	**< 0.001**
	Species richness	22.315	**< 0.001**	1	47	**< 0.001**
	True diversity (Shannon)	14.877	**< 0.001**	1	47	**< 0.001**
	True diversity (Simpson)	8.796	**0.005**	1	47	**0.004**
	FDis	0.172	0.680	1	47	0.690
	RaoQ	0.002	0.994	1	47	0.994

*Statistically significant effects (p < 0.05) are reported in bold. F, F statistic; d.f., degrees of freedom.*

**TABLE 3:** GLMM (full model) output showing main effects of environmental factors (fixed effects) on taxonomic and functional metrics of ciliate assemblages, with season and replicate as random effects.

	Assemblage parameter	Environmental parameter	*F*	*p*	d.f.	d.f corrected	Coefficient
**One-month**	Abundance	COD	0.137	0.713	1	50	–0.149
		Nitrites	3.079	0.085	1	50	3.947
	Species richness	COD	1.117	0.296	1	50	0.234
		Nitrites	0.013	0.910	1	50	–0.089
	True diversity (Shannon)	COD	0.469	0.497	1	50	0.068
		Nitrites	2.542	0.117	1	50	–0.592
	True diversity (Simpson)	COD	0.141	0.709	1	50	–0.063
		Nitrites	0.952	0.334	1	50	–0.608
	FDis	COD	1.166	0.285	1	50	–0.430
		Nitrites	0.064	0.801	1	50	0.429
	RaoQ	COD	0.732	0.396	1	50	–0.511
		Nitrites	4.497	**0.039**	1	50	–4.838
**Two-months**	Abundance	COD	5.844	**0.009**	1	43	–0.776
		Conductivity	4.059	0.050	1	43	–0.010
		pH	0.719	0.395	1	43	1.604
		Ortho	0.971	0.348	1	43	5.925
	Species richness	COD	0.547	0.464	1	43	0.173
		Conductivity	8.348	**0.006**	1	43	–0.010
		pH	3.594	0.065	1	43	1.276
		Ortho	1.223	0.275	1	43	3.340
	True diversity (Shannon)	COD	1.811	0.185	1	43	0.264
		Conductivity	8.976	**0.005**	1	43	–0.009
		pH	9.489	**0.004**	1	43	1.929
		Ortho	4.022	0.051	1	43	5.522
	True diversity (Simpson)	COD	1.714	0.197	1	43	0.234
		Conductivity	9.639	**0.003**	1	43	–0.009
		pH	12.055	**0.001**	1	43	2.021
		Ortho	4.810	**0.034**	1	43	5.685
	FDis	COD	1.462	0.233	1	43	1.693
		Conductivity	0.659	0.465	1	43	–0.019
		pH	2.076	0.071	1	43	2.954
		Ortho	1.252	0.273	1	43	13.017
	RaoQ	COD	7.844	**0.009**	1	43	3.018
		Conductivity	2.293	0.263	1	43	–0.012
		pH	3.193	0.071	1	43	4.823
		Ortho	1.496	0.107	1	43	6.979

*Statistically significant effects (p < 0.05) are reported in bold. F, F statistic; d.f., degrees of freedom.*

**TABLE 4:** GLMM (full model) output showing main effects of periphyton-associated factors (fixed effects) on taxonomic and functional metrics of ciliate assemblages, with season and replicate as random effects.

	Assemblage parameter	Community parameter	*F*	*p*	d.f.	d.f. corrected	Coefficient
**One-month**	Abundance	Organic matter	0.007	0.935	1	50	0.010
		Inorganic matter	0.732	0.396	1	50	–0.014
		Chlorophyll *a*	0.918	0.343	1	50	–0.755
	Species richness	Organic matter	0.013	0.908	1	50	–0.002
		Inorganic matter	2.254	0.140	1	50	0.014
		Chlorophyll *a*	6.250	**0.016**	1	50	–0.540
	True diversity (Shannon)	Organic matter	2.701	0.107	1	50	–0.014
		Inorganic matter	2.266	0.139	1	50	0.007
		Chlorophyll *a*	20.578	**< 0.001**	1	50	–0.259
	True diversity (Simpson)	Organic matter	2.902	0.095	1	50	–0.019
		Inorganic matter	37.675	**< 0.001**	1	50	0.005
		Chlorophyll *a*	889.41	**< 0.001**	1	50	–0.337
	FDis	Organic matter	31.786	**< 0.001**	1	50	0.141
		Inorganic matter	0.195	0.709	1	50	–0.018
		Chlorophyll *a*	0.459	0.517	1	50	–0.568
	RaoQ	Organic matter	1.490	0.228	1	50	–0.054
		Inorganic matter	3.245	0.077	1	50	0.038
		Chlorophyll *a*	10.050	**0.003**	1	50	–2.469
**Two months**	Abundance	Organic matter	13.031	**< 0.001**	1	47	0.370
		Inorganic matter	29.264	**< 0.001**	1	47	–0.036
		Chlorophyll *a*	2.762	0.103	1	47	0.219
	Species richness	Organic matter	1.727	0.195	1	47	0.090
		Inorganic matter	7.652	**0.008**	1	47	–0.011
		Chlorophyll *a*	3.034	0.088	1	47	0.182
	True diversity (Shannon)	Organic matter	1.142	0.291	1	47	0.028
		Inorganic matter	5.258	**0.026**	1	47	–0.005
		Chlorophyll *a*	0.581	0.450	1	47	0.002
	True diversity (Simpson)	Organic matter	0.393	0.534	1	47	0.038
		Inorganic matter	2.907	0.095	1	47	–0.006
		Chlorophyll *a*	0.444	0.509	1	47	0.061
	FDis	Organic matter	0.477	0.493	1	47	–0.223
		Inorganic matter	13.003	**< 0.001**	1	47	–0.045
		Chlorophyll *a*	2.099	0.154	1	47	0.330
	RaoQ	Organic matter	0.517	0.476	1	47	–0.235
		Inorganic matter	8.579	**0.005**	1	47	–0.039
		Chlorophyll *a*	3.785	0.058	1	47	0.445

*Statistically significant effects (p < 0.05) are reported in bold. Legend: F, F statistic; d.f., degrees of freedom.*

FDis values for ciliates were lower at N sites compared to C sites for both immersion periods, whereas RaoQ values were higher at N sites for the 1-month period but lower for the 2-months period (Figure 5). When tested with GLMMs including environmental and periphyton-associated factors, significant differences were confirmed only for FDis values for the 1-month period and RaoQ values for the 2-months period (Tables 1, 2). Both functional metrics for the 2-months period were negatively correlated with conductivity, while they were positively correlated with pH, COD, and orthophosphates, although the correlations were significant only for RaoQ (Table 3). As for periphyton-associated factors, FDis and RaoQ showed quite different correlations for the 1-month period: FDis was significantly positively correlated with organic matter and negatively correlated with inorganic matter, whereas RaoQ was significantly negatively correlated with organic matter and positively correlated with inorganic matter. Both functional metrics were then negatively correlated with chlorophyll *a*, while a significant correlation was found only for RaoQ. With regards to the 2-months period, both FDis and RaoQ were positively correlated with chlorophyll *a* and negatively correlated with organic and inorganic matter. Correlations with inorganic matter were the only found significant (Table 4).

The detailed GLMM report with all correlations between environmental factors and the 1 and 2-months taxonomic and functional metrics of ciliates can be found in Supplementary Table 4.

In terms of immersion duration, it could be noticed that all ciliate taxonomic metrics at N sites increased with longer immersion. The same could be observed for FDis while RaoQ values remained the same.

### Taxonomic and Functional Metrics of Amoeboid Protists

**FIGURE 6:**
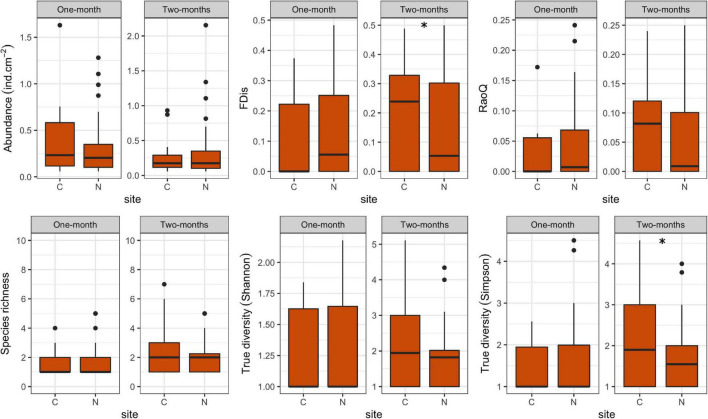
Box plots showing taxonomic and functional metrics of amoeboid protists at control (C) and revitalized sites (N) for the 1 and 2-months immersion periods. Asterix symbol (*) indicates statistically significant differences among estimated means (GLMM, *p* < 0.05). Upper and lower edges of the boxes are the first and third quartiles; the line inside the box represents the median; individual dots are outliers.

A total of 52 species of amoeboid protists were identified in this study, most of which (41) belonged to N sites. Almost no differences were observed between N and C sites with respect to most taxonomic metrics for both immersion periods (Figure 6). Abundance was slightly lower at N sites for the 1-month period while True diversity Shannon values were significantly lower at N sites for the 2-months period, when tested with GLMMs that included environmental and periphyton-associated factors (Tables 5, 6). As for correlations with environmental factors, all taxonomic metrics for the 1-month period were positively correlated with COD, while the correlation with abundance was found significant. With regards to the 2-months period, all taxonomic metrics negatively correlated with COD and orthophosphates, while a positive correlation was found with pH and conductivity. Most of these correlations were significant (Table 7). As for correlations with periphyton-associated factors, all taxonomic metrics for the 1-month period were positively correlated with all three factors, while significant correlations were found between species richness and inorganic matter. For the 2-months period, there was a significant positive correlation between abundance and organic matter, while all taxonomic metrics were significantly negatively correlated with chlorophyll *a* (Table 8).

**TABLE 5:** GLMM (full model) output showing differences in amoeboid protist assemblage metrics between control and revitalized sites (1 and 2-months immersion periods) with sites and environmental factors as fixed effects, and season and replicate as random effects.

	Assemblage parameter	*F*	*p*	d.f	d.f. corrected	p C-N
**One-month**	Abundance	0.082	0.776	1	50	0.781
	Species richness	0.009	0.925	1	50	0.924
	True diversity (Shannon)	0.345	0.560	1	50	0.554
	True diversity (Simpson)	0.482	0.491	1	50	0.479
	FDis	0.566	0.455	1	50	0.458
	RaoQ	0.607	0.440	1	50	0.428
**Two-months**	Abundance	0.661	0.421	1	43	0.406
	Species richness	0.274	0.603	1	43	0.595
	True diversity (Shannon)	0.005	0.941	1	43	0.941
	True diversity (Simpson)	0.116	0.735	1	43	0.738
	FDis	5.246	**0.027**	1	43	0.121
	RaoQ	1.743	0.194	1	43	0.282

*Statistically significant effects (p < 0.05) are reported in bold. F, F statistic; d.f., degrees of freedom.*

**TABLE 6:** GLMM (full model) output showing differences in amoeboid protist assemblage metrics between control and revitalized sites (1 and 2-months immersion periods) with sites and periphyton-associated factors as fixed effects, and season and replicate as random effects.

	Assemblage parameter	*F*	*p*	d.f	d.f. corrected	p C-N
**One-month**	Abundance	1.782	0.188	1	50	0.243
	Species richness	0.007	0.932	1	50	0.932
	True diversity (Shannon)	0.003	0.955	1	50	0.955
	True diversity (Simpson)	0.146	0.704	1	50	0.700
	FDis	1.555	0.218	1	50	0.244
	RaoQ	2.641	0.110	1	50	0.118
**Two-months**	Abundance	0.144	0.706	1	47	0.712
	Species richness	1.420	0.239	1	47	0.267
	True diversity (Shannon)	2.607	0.113	1	47	0.131
	True diversity (Simpson)	4.528	**0.039**	1	47	0.050
	FDis	1.698	0.199	1	47	0.256
	RaoQ	0.608	0.439	1	47	0.482

*Statistically significant effects (p < 0.05) are reported in bold. F, F statistic; d.f., degrees of freedom.*

**TABLE 7:** GLMM (full model) output showing main effects of environmental factors (fixed effects) on taxonomic and functional metrics of amoeboid protist assemblages, with season and replicate as random effects.

	Assemblage parameter	Environmental parameter	*F*	*p*	d.f.		Coefficient
**One-month**	Abundance	COD	4.834	**0.033**	1	50	0.690
		Nitrites	2.463	0.123	1	50	2.164
	Species richness	COD	1.629	0.208	1	50	0.169
		Nitrites	8.021	**0.007**	1	50	–3.526
	True diversity (Shannon)	COD	0.379	0.541	1	50	0.050
		Nitrites	6.716	**0.012**	1	50	–1.404
	True diversity (Simpson)	COD	0.655	0.422	1	50	0.100
		Nitrites	5.413	**0.024**	1	50	–2.208
	FDis	COD	0.146	0.704	1	50	–0.318
		Nitrites	3.805	0.057	1	50	–17.316
	RaoQ	COD	0.370	0.546	1	50	–0.437
		Nitrites	4.466	**0.040**	1	50	–15.668
**Two-months**	Abundance	COD	3.861	0.056	1	43	–0.611
		Conductivity	5.605	**0.022**	1	43	0.013
		pH	5.347	**0.026**	1	43	2.548
		Ortho	1.893	0.176	1	43	–6.440
	Species richness	COD	0.149	0.701	1	43	–0.091
		Conductivity	13.119	**< 0.001**	1	43	0.022
		pH	1.949	0.170	1	43	0.988
		Ortho	5.915	**0.019**	1	43	–8.005
	True diversity (Shannon)	COD	0.598	0.444	1	43	–0.174
		Conductivity	12.899	**< 0.001**	1	43	0.016
		pH	1.774	0.190	1	43	0.891
		Ortho	5.127	**0.029**	1	43	–6.502
	True diversity (Simpson)	COD	1.051	0.311	1	43	–0.217
		Conductivity	13.285	**< 0.001**	1	43	0.016
		pH	1.276	0.265	1	43	0.707
		Ortho	5.754	**0.021**	1	43	–6.580
	FDis	COD	4.779	**0.034**	1	43	–1.534
		Conductivity	0.523	0.473	1	43	–0.009
		pH	11.541	**0.001**	1	43	7.378
		Ortho	6.533	**0.014**	1	43	23.133
	RaoQ	COD	0.522	0.474	1	43	–0.526
		Conductivity	0.042	0.839	1	43	–0.003
		pH	3.950	0.053	1	43	4.859
		Ortho	3.839	0.057	1	43	19.350

*Statistically significant effects (p < 0.05) are reported in bold. F, F statistic; d.f., degrees of freedom.*

**TABLE 8:** GLMM (full model) output showing main effects of periphyton-associated factors (fixed effects) on taxonomic and functional metrics of amoeboid protist assemblages, with season and replicate as random effects.

One-month	Assemblage parameter	Community parameter	*F*	*p*	d.f.	d.f. corrected	Coefficient
	Abundance	Organic matter	0.001	0.981	1	50	0.001
		Inorganic matter	0.007	0.935	1	50	–0.001
		Chlorophyll *a*	2.170	0.147	1	50	0.558
	Species richness	Organic matter	2.530	0.118	1	50	0.030
		Inorganic matter	5.245	**0.026**	1	50	0.020
		Chlorophyll *a*	3.568	0.065	1	50	0.558
	True diversity (Shannon)	Organic matter	0.012	0.915	1	50	0.001
		Inorganic matter	0.001	0.969	1	50	0.001
		Chlorophyll *a*	0.001	0.972	1	50	0.001
	True diversity (Simpson)	Organic matter	1.707	0.197	1	50	0.028
		Inorganic matter	3.318	0.075	1	50	0.014
		Chlorophyll *a*	1.907	0.173	1	50	0.375
	FDis	Organic matter	0.733	0.396	1	50	0.061
		Inorganic matter	7.863	**0.007**	1	50	0.087
		Chlorophyll *a*	0.001	0.980	1	50	0.025
	RaoQ	Organic matter	1.612	0.210	1	50	0.080
		Inorganic matter	7.828	**0.007**	1	50	0.072
		Chlorophyll *a*	0.363	0.550	1	50	0.446
**Two months**	Abundance	Organic matter	6.653	**0.013**	1	47	0.185
		Inorganic matter	2.537	0.118	1	47	–0.006
		Chlorophyll *a*	17.245	**< 0.001**	1	47	–0.736
	Species richness	Organic matter	0.022	0.882	1	47	0.008
		Inorganic matter	3.885	0.055	1	47	0.005
		Chlorophyll *a*	21.774	**< 0.001**	1	47	–0.460
	True diversity (Shannon)	Organic matter	1.552	0.219	1	47	0.070
		Inorganic matter	0.390	0.535	1	47	0.002
		Chlorophyll *a*	12.681	**< 0.001**	1	47	–0.297
	True diversity (Simpson)	Organic matter	2.197	0.145	1	47	0.074
		Inorganic matter	0.412	0.524	1	47	0.002
		Chlorophyll *a*	11.839	**0.001**	1	47	–0.261
	FDis	Organic matter	0.051	0.822	1	47	0.037
		Inorganic matter	1.616	0.210	1	47	0.011
		Chlorophyll *a*	1.078	0.304	1	47	–0.282
	RaoQ	Organic matter	0.220	0.642	1	47	0.115
		Inorganic matter	0.640	0.428	1	47	0.010
		Chlorophyll *a*	1.096	0.300	1	47	–0.392

*Statistically significant effects (p < 0.05) are reported in bold. F, F statistic; d.f., degrees of freedom.*

FDis and RaoQ values were higher at N sites compared with C sites for the 1-month period, whereas they were lower for the 2-months period (Figure 6). The only significant difference was found for the 1-month period. Regarding correlations with environmental factors, both functional indices were negatively correlated with COD for both immersion periods, while significant correlations were found only for the 2-months period. In addition, FDis was significantly positively correlated with pH and orthophosphates (Table 7). As for periphyton-associated factors, both functional indices were positively correlated with all periphyton-associated factors for the 1-month period, whereas they were negatively correlated with chlorophyll *a* for the 2-months period, although not significantly (Table 8).

The detailed GLMM report with all correlations between environmental factors and the 1 and 2-months taxonomic and functional metrics of amoeboid protists can be found in Supplementary Table 5.

With respect to immersion duration, species richness and True diversity (Shannon and Simpson) values at N sites were found to increase with longer immersion, while abundance decreased slightly. The functional indices remained more or less the same with longer immersion duration.

## Discussion

Our results revealed that ciliates and amoeboid protists respond differently to stream revitalization at the assemblage level. While ciliate assemblages at N sites had higher abundance and taxonomic diversity compared to those at C sites for both immersion periods, taxonomic metrics of amoeboid assemblages differed little between sites, even with longer immersion. The two protist assemblages also differed in their functional response: while the functional diversity of ciliates was lower at N sites compared to C sites for both immersion periods, that of amoeboid protists was higher at N sites for the 1-month period but lower for the 2-months period, in comparison to C sites.

The lower functional diversity of ciliates at N sites compared to C sites for both immersion periods suggests that the level of disturbance in reactivated streams, in the form of occasionally changing hydrologic conditions (high flow or drought), was either too high or too low to promote functional diversity, judging by the intermediate disturbance hypothesis (IDH). IDH rests on the assumption that a maximum level of diversity is achieved when the assemblage is exposed to an intermediate level of disturbance (Connell, 1978). It is likely that in our case, disturbance filtered out incompatible suites of traits, so that only a subset of species with disturbance-resistant traits could colonize a disturbed habitat (Lavorel et al., 2008; Biswas and Mallik, 2010). Thus, compared to C sites, ciliate assemblages at N sites could be expected to consist mainly of prospective colonizers, with disturbance-intolerant species (r-strategists) absent or their share in the assemblage minimized, leading to increased similarity and functional redundancy within the assemblage (Segovia et al., 2016). On the other hand, it appears that amoeboid assemblages at N sites during the 1-month period consisted mainly of highly tolerant or even drought-resistant species (K-strategists) that would thrive under hydrologically extreme conditions (Carballeira and Pontevedra-Pombal, 2021), in contrast to the r-strategists that probably dominated the 2-months period, leading to differences in functional diversity between the two periods.

Our results suggested that the reasons for these different responses to stream revitalization in terms of taxonomic and functional metrics could also lie in the effects of environmental and periphyton-associated factors. While some factors had a similar effect on both assemblages, others affected the assemblages quite differently. The overall observed positive effect of COD, pH, and organic matter content on taxonomic metrics of both ciliates and amoeboid protists is consistent with previous findings by numerous authors who have worked with these protists (Cowling, 1994; Smith, 1996; Verhoeven, 2002; Bates et al., 2013; Bradford, 2016; Geisen, 2016; Fiore-Donno et al., 2019). The likely reason for this is the increased diversity and quantity of bacteria that are their main food source, as the deposited organic material supports attachment of bacteria that decompose both dissolved and particulate organic matter (Foissner, 2014). However, the dominance of bacteria as a food source could have led to functional homogeneity and explain why functional diversity of both ciliates and amoeboid protists was negatively affected by COD and/or organic matter.

Inorganic material, i.e., tufa, on the other hand, had a different effect. While it positively affected the taxonomic and functional metrics of both protist groups for the 1-month period, the same was not observed for the 2-months period. It appears that the higher inorganic matter content during the 2-months period, whose deposition was facilitated by slightly higher temperature, conductivity, and flow velocity at N sites (Supplementary Table 3), negatively affected the ciliate taxonomic and functional metrics, but did not cause any negative effects on those of the amoeboid assemblages. Although the presence of inorganic matter can increase microhabitat heterogeneity (Beisel et al., 2000; Bednar et al., 2017; Singer et al., 2021) and thus serve as an “inoculum” for periphyton supporting high abundance and diversity (Primc and Habdija, 1987; Wörner et al., 2000; Zimmermann-Timm, 2002), there appears to be a critical point at which inorganic matter content no longer promotes high abundance and taxonomic diversity of ciliates. It could be that ciliates are much more sensitive to burial and sloughing than amoeboid protists because they do not predominantly glide along the substrate as amoebae do but exhibit various modes of locomotion: many of them are free-swimmers or even move by jumping and rotating (Foissner and Berger, 1996; Esteban and Fenchel, 2021). Amoeboid protists (especially naked amoebae) are also smaller in size compared to ciliates, which allows them to savor the microhabitats and be very closely associated with the substrate (Preston, 2003).

The two assemblages also responded differently with respect to immersion duration: while ciliate taxonomic and functional metrics at N sites increased with longer immersion, the same was observed only for some taxonomic metrics of amoeboid protists (species richness and True diversity values), while the abundance decreased, and functional metrics remained more or less the same. Since chloropyhll *a* concentration showed significant correlations with most taxonomic and functional metrics of both assemblages and reached extreme values at times during the 2-months period at N sites, it is reasonable to assume that the different responses to immersion duration could be due to this. Although the observed orthophosphate levels during the 2-months period were still quite low and within the range characteristic of tufa-depositing streams (Primc-Habdija et al., 2005), they may have caused algal proliferation and subsequently higher chlorophyll *a* concentration. The dense algal coating of the substrate may have contributed to heterogeneity of food sources and microhabitats (Algarte et al., 2017) which together led to niche diversification, an increase in the share of disturbance-resistant ciliate species and subsequently an increase in taxonomic and functional diversity. Another possible explanation could be that changing hydrological conditions at N sites activate the dormant part of the microbial assemblages, leading to increased functional diversity over time (Velasco-González et al., 2020). On the other hand, the alginate covering the bacteria likely triggered a different response in the amoeboid assemblages, leading to reduced or even absent uptake of bacteria by amoeboid protists. This is likely due to the alginate coating increasing the overall size of the bacterial particles or altering their “flavor” (Heaton et al., 2001; Parry, 2004), resulting in reduced abundance of amoeboid protists during the 2-months immersion period.

The taxonomic and functional diversity of protists is a valuable indication of environmental conditions and ecosystem stability. Understanding the factors that structure protist assemblages is a prerequisite for using these organisms to predict environmental quality and future changes in ecosystem functioning. Protists have been used in environmental monitoring, but only to a limited extent because of the difficulties associated with sophisticated morphological identification methods. However, comprehensive studies that also consider their functional traits (Fournier et al., 2012) could make them more recognizable and increase the frequency of using protists as indicators of ecosystem functioning.

## Conclusion

Linking taxonomic and functional metrics to environmental conditions can improve our understanding of biological processes, especially in ecosystems undergoing extensive changes, such as revitalized streams. Our results showed that taxonomic and functional metrics of ciliates and amoeboid protists responded to the prevailing conditions characteristic of revitalized tufa-depositing streams: changing hydrology (occasional high flow or drought), soil drainage, and extensive inorganic matter, i.e., tufa deposition, although their responses were somewhat different. While both protist assemblages benefited from COD and the presence of organic matter supported by favorable pH, as this provided their main food source–bacteria, ciliates were more sensitive to the presence of inorganic matter content than amoeboid protists. However, in terms of chlorophyll *a* concentration, ciliates appeared to benefit more from the algal coating as it resulted in heterogeneity of food sources and microhabitats, which increased their taxonomic and functional diversity, while it decreased the uptake of bacteria for amoeboid protists, negatively affecting their abundance. The two assemblages also showed different responses of taxonomic and functional metrics with respect to immersion duration: while the taxonomic and functional diversity of ciliates at N sites increased with longer immersion, indicating niche diversification, those of amoeboid protists hardly changed with time. The results presented suggest that a comprehensive analysis of taxonomic and functional metrics of ciliates and amoeboid protists could be a good proxy for assessing revitalization of tufa-depositing streams. However, the temporal component should always be considered when conducting such studies, as the colonization processes of ciliates and amoeboid protists are quite complex, especially in tufa-depositing streams.

## Data Availability Statement

The original contributions presented in the study are included in the article/Supplementary Material, further inquiries can be directed to the corresponding author/s.

## Author Contributions

VG, MSP, and RMK contributed to conception and design of the study. VG and BV organized the trait database. VG and FR performed the statistical analysis. VG wrote the first draft of the manuscript. All authors contributed to manuscript revision, read, and approved the submitted version.

## Conflict of Interest

The authors declare that the research was conducted in the absence of any commercial or financial relationships that could be construed as a potential conflict of interest.

## Publisher’s Note

All claims expressed in this article are solely those of the authors and do not necessarily represent those of their affiliated organizations, or those of the publisher, the editors and the reviewers. Any product that may be evaluated in this article, or claim that may be made by its manufacturer, is not guaranteed or endorsed by the publisher.
