# Supplementary material for: Taxonomic and Functional Metrics of Ciliates and Amoeboid Protists in Response to Stream Revitalization
Source: Front Microbiol. 2022 Apr 1;13:842395. doi: 10.3389/fmicb.2022.842395 (PMC9010972; doi:10.3389/fmicb.2022.842395)
Supplement: Supplementary file 1 [file Table_1.DOCX]

**Supplementary table S1.** Functional traits of periphytic ciliate species sampled at Skradinski buk tufa barrier (Krka National Park, Croatia).

| **Taxa** | **Food source** | **Feeding strategy** | **Ecosystem preference^1^** | **Habitat preference^2^** | **Motility** | **Mode of locomotion** | **Life form** |
| --- | --- | --- | --- | --- | --- | --- | --- |
| *Acineria uncinata* Tucolesco, 1962 | phagotrophic protists, small metazoans | predation | active sludge | benthos, periphyton | motile | crawling | solitary |
| *Acineta flava* Kellicott 1885 | phagotrophic protists, small metazoans | predation | lentic and lotic (freshwater) | periphyton | sessile | unknown | solitary |
| *Acineta tuberosa* Ehrenberg, 1833 | phagotrophic protists, small metazoans | predation | active sludge | periphyton | sessile | unknown | solitary |
| *Amphileptus sp.* | phagotrophic protists, small metazoans | predation | active sludge | periphyton | motile | free-swimming | solitary |
| *Aspidisca cicada* O. F. Müller, 1786. | bacteria | filtration | active sludge | benthos, periphyton | motile | jumping, rotating | solitary |
| *Aspidisca lynceus* O.F. Müller, 1773 | bacteria | filtration | active sludge | benthos, periphyton | motile | jumping, rotating | solitary |
| *Aspidisca* sp. | bacteria | filtration | active sludge | benthos, periphyton | motile | jumping, rotating | solitary |
| *Chilodonella caudata* Stokes, 1885 | bacteria | filtration | active sludge | benthos, periphyton | motile | gliding | solitary |
| *Chilodonella uncinata* (Ehrenberg, 1838) | bacteria | filtration | active sludge | benthos, periphyton | motile | gliding | solitary |
| *Chlamydonella alpestris* Foissner, 1979 | bacteria, diatoms | predation | soil | benthos, periphyton | motile | gliding | solitary |
| *Chlamydonellopsis plurivacuolata* Blatterer & Foissner, 1990 | diatoms | predation | lentic and lotic (freshwater) | benthos, periphyton | motile | crawling | solitary |
| *Chlamydonellopsis* sp. | diatoms | predation | lentic and lotic (freshwater) | benthos, periphyton | motile | crawling | solitary |
| *Cinetochilum margaritaceum* Perty, 1852 | algae, bacteria | filtration | active sludge | planktonic | motile | free-swimming | solitary |
| *Colpoda cucullus* O.F. Müller, 1786 | omnivorous | filtration | soil | benthos | motile | free-swimming | solitary |
| *Colpoda inflata* Stokes, 1885 | algae, bacteria | filtration | soil | benthos | motile | free-swimming | solitary |
| *Colpoda steini* Maupas, 1883 | bacteria | filtration | soil | planktonic | motile | free-swimming | solitary |
| *Cyclidium glaucoma* O. F. Müller, 1773) | bacteria | filtration | active sludge | planktonic | motile | jumping, rotating | solitary |
| *Cyclidium* sp. | bacteria | filtration | active sludge | planktonic | motile | jumping, rotating | solitary |
| *Dysteria fluviatilis* (Stein, 1859) Blochmann, 1895 | bacteria | predation | lentic and lotic (freshwater) | benthos, periphyton | motile | crawling | solitary |
| *Dysteria* sp. | bacteria | predation | lentic and lotic (freshwater) | benthos, periphyton | motile | crawling | solitary |
| *Frontonia acuminata* Ehrenberg, 1833 | omnivorous | filtration | soil | planktonic | motile | free-swimming | solitary |
| *Frontonia atra* Ehrenberg, 1834 | diatoms | filtration | lentic and lotic (freshwater) | benthos, periphyton | motile | free-swimming | solitary |
| *Gastrostyla mystacea* (Stein, 1859) Sterkl, 1878 | omnivorous | filtration | soil | benthos | motile | gliding | solitary |
| *Gastrostyla steini* Hemberger, 1982 | omnivorous | filtration | soil | benthos | motile | gliding | solitary |
| *Halteria chlorelligera* Kahl, 1932 | algae | filtration | lentic and lotic (freshwater) | planktonic | motile | jumping | solitary |
| *Holophrya discolor* Ehrenberg, 1833 | omnivorous | predation | active sludge | benthos, periphyton | motile | free-swimming | solitary |
| *Holophrya ovum* Ehrenberg, 1831 | omnivorous | predation | lentic and lotic (freshwater) | benthos, periphyton | motile | free-swimming | solitary |
| *Holophrya* sp*.* | omnivorous | predation | active sludge | planktonic | motile | free-swimming | solitary |
| *Holophrya teres* Ehrenberg, 1834 | omnivorous | predation | lentic and lotic (freshwater) | planktonic | motile | free-swimming | solitary |
| *Holosticha kessleri* (Wrześniowski, 1877) Šrámek-Hušek, 1957 | bacteria | filtration | lentic and lotic (freshwater) | benthos | motile | crawling | solitary |
| *Holosticha multistilata* Kahl, 1928 | omnivorous | filtration | lentic and lotic (freshwater) | benthos | motile | free-swimming | solitary |
| *Holosticha pullaster* (Müller, 1773) Foissner, Blatterer, Berger & Kohmann, 1991 | omnivorous | filtration | soil | benthos | motile | free-swimming | solitary |
| *Lacrymaria olor* O. F. Müller, 1776 | phagotrophic protists, small metazoans | predation | lentic and lotic (freshwater) | benthos, periphyton | motile | free-swimming | solitary |
| *Lembadion bullinum* (Müller, 1786) Perty, 1849 | omnivorous | filtration | lentic and lotic (freshwater) | benthos | motile | free-swimming | solitary |
| *Lembadion* sp*.* | omnivorous | filtration | lentic and lotic (freshwater) | benthos | motile | free-swimming | solitary |
| *Leptopharynx costatus* Mermod, 1914 | algae, bacteria | predation | soil | planktonic | motile | crawling | solitary |
| *Litonotus cygnus* (O.F. Müller, 1776) Wrzesniowski, 1870 | phagotrophic protists, small metazoans | predation | lentic and lotic (freshwater) | benthos, periphyton | motile | crawling | solitary |
| *Litonotus lamella* Schewiakoff, 1896 | phagotrophic protists, small metazoans | predation | lentic and lotic (freshwater) | benthos, periphyton | motile | crawling | solitary |
| *Litonotus* sp. | phagotrophic protists, small metazoans | predation | lentic and lotic (freshwater) | benthos, periphyton | motile | crawling | solitary |
| *Loxophyllum utriculariae* Penard, 1922 | phagotrophic protists, small metazoans | predation | lentic and lotic (freshwater) | periphyton | motile | crawling | solitary |
| *Nassula picta* Greeff, 1888 | cyanobacteria | predation | soil | planktonic | motile | crawling | solitary |
| *Oxytricha chlorelligera* Kahl, 1932 | omnivorous | filtration | lentic and lotic (freshwater) | benthos, periphyton | motile | free-swimming | solitary |
| *Oxytricha ferruginea* Stein, 1859 | omnivorous | filtration | lentic and lotic (freshwater) | benthos | motile | free-swimming | solitary |
| *Oxytricha setigera* Stokes, 1891 | bacteria | filtration | soil | benthos | motile | free-swimming | solitary |
| *Oxytricha* sp. | omnivorous | filtration | lentic and lotic (freshwater) | benthos | motile | crawling | solitary |
| *Paraurostyla viridis* (Stein, 1859) Borror, 1972 | bacteria | filtration | lentic (freshwater) | benthos | motile | free-swimming | solitary |
| *Paraurostyla weissei* (Stein, 1859) Borror, 1972 | omnivorous | filtration | lentic and lotic (freshwater) | benthos | motile | crawling | solitary |
| *Phialina* sp. | phagotrophic protists, small metazoans | predation | soil | benthos, periphyton | motile | free-swimming | solitary |
| *Plagiocampa rouxi* Kahl, 1926 | algae, bacteria | predation | soil | planktonic | motile | free-swimming | solitary |
| *Platyophrya vorax* Kahl, 1926 | omnivorous | predation | soil | benthos | motile | free-swimming | solitary |
| *Pleurotricha grandis* Stein, 1859 | algae, diatoms | filtration | lentic and lotic (freshwater) | benthos | motile | crawling | solitary |
| *Pseudochilodonopsis algivora* (Kahl, 1931) Foissner, 1979 | algae, bacteria | predation | lentic and lotic (freshwater) | planktonic | motile | unknown | solitary |
| *Pseudochilodonopsis fluviatilis* Foissner, 1988 | diatoms | predation | active sludge | benthos, periphyton | motile | crawling | solitary |
| *Pseudochilodonopsis* sp. | algae | predation | lentic and lotic (freshwater) | planktonic | motile | crawling | solitary |
| *Pseudocohnilembus pusillus* (Quennerstedt, 1869) Foissner & Wilbert, 1981 | bacteria | filtration | soil | benthos, periphyton | motile | crawling | solitary |
| *Pseudovorticella* sp*.* | algae, bacteria | filtration | soil | benthos, periphyton | semi-sessile | unknown | colonial |
| *Rimostrombidium lacustris* (Foissner, Skogstad & Pratt, 1988) Petz & Foissner, 1992 | algae, bacteria | filtration | lentic (freshwater) | planktonic | motile | jumping | solitary |
| *Rimostrombidium* sp*.* | algae, bacteria | filtration | lentic (freshwater) | planktonic | motile | jumping | solitary |
| *Spathidium* sp. | cyanobacteria | predation | soil | planktonic | motile | free-swimming | solitary |
| *Spirostomum ambiguum* (Müller, 1786) Ehrenberg, 1835 | omnivorous | filtration | lentic and lotic (freshwater) | benthos, periphyton | motile | free-swimming | solitary |
| *Stentor roeselii* Ehrenberg, 1835 | omnivorous | filtration | lentic and lotic (freshwater) | benthos, periphyton | semi-sessile | unknown | solitary |
| *Stentor* sp. | omnivorous | filtration | lentic and lotic (freshwater) | benthos, periphyton | motile | free-swimming | solitary |
| *Sterkiella histriomuscorum* Foissner, Blatterer, Berger & Kohmann, 1991 | omnivorous | predation | soil | benthos | motile | free-swimming | solitary |
| *Tetrahymena pyriformis* (Ehrenberg, 1830) Furgason, 1940 | bacteria | filtration | active sludge | benthos | motile | free-swimming | solitary |
| *Thigmogaster* sp. | bacteria | predation | lentic and lotic (freshwater) | benthos, periphyton | motile | gliding | solitary |
| *Trithigmostoma cucullulus* Jankowski, 1967 | omnivorous | predation | soil | benthos, periphyton | motile | crawling | solitary |
| *Trochilioides recta* Kahl, 1928 | bacteria | predation | lentic and lotic (freshwater) | benthos, periphyton | motile | free-swimming | solitary |
| *Uroleptus musculus* O. F. Muller, 1773 | omnivorous | filtration | lentic and lotic (freshwater) | benthos, periphyton | motile | free-swimming | solitary |
| *Uroleptus piscis* (O. F. Müller, 1773) Ehrenberg, 1831 | omnivorous | filtration | lentic and lotic (freshwater) | benthos, periphyton | motile | crawling | solitary |
| *Uronema nigricans* O. F. Müller, 1786) Florentin, 1901 | omnivorous | filtration | lentic and lotic (freshwater) | planktonic | motile | rotating | solitary |
| *Urostyla grandis* Ehrenberg, 1830 | omnivorous | predation | lentic and lotic (freshwater) | benthos | motile | free-swimming | solitary |
| *Urotricha* sp. | algae, bacteria | filtration | lentic and lotic (freshwater) | planktonic | motile | free-swimming | solitary |
| *Vorticella aquadulcis,* Stokes, 1887 | algae, bacteria | filtration | active sludge | benthos, periphyton | semi-sessile | unknown | colonial |
| *Vorticella convallaria-complex* Linnaeus, 1758 | bacteria | filtration | active sludge | benthos, periphyton | semi-sessile | unknown | colonial |
| *Vorticella infusionum-complex* Dujardin, 1841 | bacteria | filtration | soil | benthos, periphyton | semi-sessile | unknown | colonial |
| *Vorticella octava-complex* Stokes, 1885 | bacteria | filtration | active sludge | periphyton | semi-sessile | unknown | colonial |
| *Vorticella* sp*.* | omnivorous | filtration | active sludge | periphyton | semi-sessile | unknown | colonial |

^1^Ecosystem preference refers to the type of ecosystem in which the species preferentially occurs. Species labelled "soil" or "active sludge" also occur in soil and active sludge in addition to freshwater ecosystems (lentic and lotic).

^2^Habitat preference refers to the type of habitat in which the species preferentially occurs. Species labelled "planktonic" also occur in plankton in addition to benthos and periphyton.
