# Supplementary material for: Taxonomic and Functional Metrics of Ciliates and Amoeboid Protists in Response to Stream Revitalization
Source: Front Microbiol. 2022 Apr 1;13:842395. doi: 10.3389/fmicb.2022.842395 (PMC9010972; doi:10.3389/fmicb.2022.842395)
Supplement: Supplementary file 2 [file Table_2.DOCX]

**Supplementary table S2.** Functional traits of periphytic amoeboid protist species sampled at Skradinski buk tufa barrier (Krka National Park, Croatia).

| **Taxa** | **Food source** | **Habitat** | **Morphology** |
| --- | --- | --- | --- |
| *Amoeba proteus* (Pallas, 1766) Leidy, 1878 | protists | freshwater | naked amoeba |
| *Arcella* sp. | protists | freshwater | naked amoeba |
| *Chaos* sp*.* | diatoms | mosses | naked amoeba |
| *Chlamydophrys minor* Belar, 1921 | unknown | freshwater | testate amoeba |
| *Cyclopyxis kahli* Deflandre, 1929 | bacteria | soil | testate amoeba |
| *Dermamoeba* sp. | cyanobacteria | soil | naked amoeba |
| *Discamoeba* sp. | bacteria | mosses | testate amoeba |
| *Euglypha bryophila* Brown, 1911 | bacteria | mosses | testate amoeba |
| *Euglypha cristata* Leidy, 1874 | bacteria | freshwater | naked amoeba |
| *Euglypha* laevis Perty, 1849 | omnivorous | mosses | testate amoeba |
| *Filamoeba nolandi* Page, 1967 | bacteria | freshwater | naked amoeba |
| *Filamoeba* sp*.* | bacteria | freshwater | naked amoeba |
| *Flamella* sp*.* | omnivorous | soil | testate amoeba |
| *Hartmanella* sp*.* | bacteria | freshwater | naked amoeba |
| *Heterophrys*sp. | bacteria | freshwater | naked amoeba |
| *Korotnevella* sp. | bacteria | soil | naked amoeba |
| *Lecythium hyalinum* (Ehrenberg 1838) Hertwig and Lesser 1874 | bacteria | freshwater | naked amoeba |
| *Lecythium terrestris* | algae, fungi | soil | testate amoeba |
| *Mayorella augusta* Schaeffer, 1926 | omnivorous | soil | naked amoeba |
| *Mayorella penardi* Page, 1972 | omnivorous | soil | naked amoeba |
| *Mayorella* sp*.* | omnivorous | soil | naked amoeba |
| *Mayorella viridis* Leidy, 1874 | omnivorous | mosses | naked amoeba |
| *Nuclearia radians* (Greeff 1869) Patterson 1984 | bacteria | freshwater | naked amoeba |
| *Nuclearia simplex* Cienkowsky 1865 | bacteria | freshwater | naked amoeba |
| *Nuclearia* sp*.* | bacteria | freshwater | naked amoeba |
| *Parachaos* sp*.* | protists | freshwater | naked amoeba |
| *Paradermamoeba sp.* | bacteria | freshwater | naked amoeba |
| *Penardia mutabilis* Cash, 1904 | bacteria | soil | naked amoeba |
| *Platyamoeba sp.* | bacteria | soil | naked amoeba |
| *Polychaos annulatum* (Penard, 1902) Smirnov & Goodkov, 1997 | omnivorous | soil | naked amoeba |
| *Polychaos dubium* (Schaeffer, 1916) | omnivorous | soil | naked amoeba |
| *Polychaos fasciculatum* (Penard, 1902) Schaeffer 1926 | protists | soil | naked amoeba |
| *Polychaos* sp*.* | protists | soil | naked amoeba |
| *Raphidiophrys* sp*.* | bacteria | soil | naked amoeba |
| *Reticulomyxa filosa* Nauss 1949 | bacteria | freshwater | naked amoeba |
| *Saccamoeba limax* Dujardin, 1841 | bacteria | freshwater | naked amoeba |
| *Saccamoeba lucens* Frenzel, 1892 | bacteria | freshwater | naked amoeba |
| *Saccamoeba* sp. | bacteria | freshwater | naked amoeba |
| *Thecamoeba quadrilineata* (Carter, 1856) Lepşi, 1960 | bacteria | soil | naked amoeba |
| *Thecamoeba similis* (Greeff, 1891) Lepşi, 1960 | bacteria | soil | naked amoeba |
| *Thecamoeba* sp. | bacteria | freshwater | naked amoeba |
| *Thecamoeba terricola* (Greeff,1866) Lepsi, 1960 | omnivorous | mosses | testate amoeba |
| *Trachelocorythion pulchellum* Penard, 1890 | bacteria | freshwater | naked amoeba |
| *Trichamoeba sinuosa* Siemensma & Page, 1986 | bacteria | freshwater | naked amoeba |
| *Trichamoeba* sp*.* | bacteria | soil | testate amoeba |
| *Trinema enchelys* (Ehrenberg, 1938) Leidy, 1878 | bacteria | soil | testate amoeba |
| *Trinema lineare* Penard, 1890 | bacteria | soil | testate amoeba |
| *Vahlkampfia* sp*.* | bacteria | freshwater | naked amoeba |
| *Vahlkampfia tachypodia* Glaser, 1912 | bacteria | freshwater | naked amoeba |
| *Vannella simplex* Bovee, 1965 | bacteria | freshwater | naked amoeba |
| *Vannella* sp. | bacteria | freshwater | naked amoeba |
