# Supplementary material for: Taxonomic and Functional Metrics of Ciliates and Amoeboid Protists in Response to Stream Revitalization
Source: Front Microbiol. 2022 Apr 1;13:842395. doi: 10.3389/fmicb.2022.842395 (PMC9010972; doi:10.3389/fmicb.2022.842395)
Supplement: Supplementary file 3 [file Table_3.DOCX]

**Supplementary table S3.** Values of all environmental factors measured during the research period.

One-month immersion period

| Site | Season | Temp (°C) | O_2_  (mg L^-1^) | Flow velocity  (ms^-1^) | Conductivity  (μS cm^-1^) | pH | COD KMnO_4_  (mg O_2_ L^-1^) | Alkalinity  (mg CaCO_3_ L-^1^) | Total  water  hardness  (mg CaCO_3_ L^-1^) | N-NO_2_^-^  (mg L^-1^) | N-NO_3_^-^  (mg L^-1^) | P-PO_4_^3-^  (mg L^-1^) |
| --- | --- | --- | --- | --- | --- | --- | --- | --- | --- | --- | --- | --- |
| C1 | Spring | 21.60 | 8.79 | 0.59 | 345.00 | 8.24 | 1.79 | 80.00 | 128.16 | 0.1752 | 6.9471 | 0.0545 |
| C1 | Summer | 22.80 | 8.46 | 0.35 | 330.00 | 8.23 | 1.41 | 110.00 | 258.10 | 0.0712 | 0.5000 | 0.0112 |
| C1 | Autumn | 21.00 | 8.51 | 0.37 | 296.00 | 8.18 | 2.04 | 125.00 | 320.40 | 0.1016 | 0.2647 | 0.0112 |
| C1 | Winter | 10.80 | 10.84 | 0.53 | 387.00 | 8.57 | 2.08 | 110.00 | 382.70 | 0.2792 | 6.2118 | 2.0822 |
| C2 | Spring | 21.90 | 8.71 | 1.23 | 343.00 | 8.21 | 0.39 | 75.00 | 165.54 | 0.1616 | 6.9118 | 0.0556 |
| C2 | Summer | 22.80 | 8.48 | 0.98 | 333.00 | 8.25 | 1.49 | 115.00 | 158.42 | 0.0680 | 0.3000 | 0.0309 |
| C2 | Autumn | 20.80 | 8.98 | 0.71 | 290.00 | 8.05 | 2.51 | 135.00 | 311.50 | 0.1232 | 0.3176 | 0.0146 |
| C2 | Winter | 10.50 | 10.94 | 0.69 | 390.00 | 8.75 | 1.89 | 95.00 | 373.80 | 0.2776 | 6.6529 | 1.8858 |
| N1 | Spring | 21.60 | 8.56 | 0.56 | 347.00 | 8.37 | 0.78 | 95.00 | 178.00 | 0.1616 | 5.0647 | 0.0382 |
| N1 | Summer | 22.70 | 8.38 | 0.63 | 325.00 | 8.37 | 1.10 | 70.00 | 306.16 | 0.0632 | 0.3471 | 0.0197 |
| N1 | Autumn | 20.70 | 9.04 | 0.78 | 283.00 | 8.20 | 2.12 | 145.00 | 391.60 | 0.1112 | 0.3412 | 0.0140 |
| N1 | Winter | 10.80 | 10.79 | 0.92 | 383.00 | 8.57 | 1.81 | 100.00 | 356.00 | 0.2832 | 6.4100 | 1.8070 |
| N2 | Spring | 21.40 | 8.59 | 0.39 | 335.00 | 8.33 | 0.86 | 110.00 | 160.20 | 0.1752 | 5.9647 | 0.0348 |
| N2 | Summer | 22.60 | 8.39 | 0.41 | 319.00 | 8.08 | 1.26 | 85.00 | 169.10 | 0.0712 | 0.3588 | 0.0140 |
| N2 | Autumn | 20.60 | 9.00 | 0.58 | 291.00 | 8.18 | 2.75 | 130.00 | 436.10 | 0.1176 | 0.2588 | 0.0180 |
| N2 | Winter | 10.70 | 10.86 | 0.40 | 375.00 | 8.61 | 1.89 | 77.50 | 373.80 | 0.2848 | 6.9350 | 1.8850 |
| N3 | Spring | 20.60 | 8.62 | 0.96 | 341.00 | 8.16 | 0.54 | 100.00 | 188.68 | 0.1640 | 6.5294 | 0.0556 |
| N3 | Summer | 22.60 | 8.43 | 0.92 | 442.00 | 8.08 | 1.34 | 85.00 | 151.30 | 0.0200 | 0.3882 | 0.0185 |
| N3 | Autumn | 20.60 | 8.74 | 0.68 | 254.00 | 8.13 | 2.04 | 125.00 | 293.70 | 0.1088 | 0.2882 | 0.0163 |
| N3 | Winter | 10.60 | 10.90 | 1.73 | 378.00 | 8.61 | 1.96 | 95.00 | 338.20 | 0.2816 | 6.2940 | 1.9640 |
| N4 | Spring | 20.60 | 8.82 | 1.35 | 349.00 | 8.22 | 0.39 | 80.00 | 211.82 | 0.1672 | 7.4235 | 0.0326 |
| N4 | Summer | 22.60 | 8.51 | 0.99 | 320.00 | 8.06 | 1.10 | 110.00 | 249.20 | 0.0696 | 0.4471 | 0.0101 |
| N4 | Autumn | 21.10 | 8.87 | 1.01 | 280.00 | 8.16 | 2.04 | 105.00 | 409.40 | 0.0872 | 0.2118 | 0.0146 |
| N4 | Winter | 10.40 | 10.99 | 1.39 | 378.00 | 8.60 | 2.04 | 117.50 | 356.00 | 0.2776 | 6.7294 | 2.0429 |
| N5 | Spring | 21.70 | 8.63 | 0.63 | 352.00 | 8.23 | 0.39 | 90.00 | 181.56 | 0.1552 | 6.9882 | 0.0287 |
| N5 | Summer | 22.80 | 8.42 | 0.23 | 327.00 | 8.20 | 1.34 | 115.00 | 142.40 | 0.0712 | 0.3294 | 0.0202 |
| N5* | Autumn |  |  |  |  |  |  |  |  |  |  |  |
| N5 | Winter | 10.90 | 10.76 | 0.91 | 382.00 | 8.71 | 1.96 | 105.00 | 391.60 | 0.2808 | 6.7294 | 1.9643 |

*N5 Autumn - dry

Two-months immersion period

| Site | Season | Temp (°C) | O_2_ (mgL^-1^) | Flow velocity  (ms^-1^) | Conductivity (μS cm^-1^) | pH | COD KMnO_4_  (mg O_2_ L^-1^) | Alkalinity  (mg CaCO_3_ L^-1^) | Total  water  hardness  (mg CaCO_3_ L^-1^) | N-NO_2_^-^ (mg L^-1^) | N-NO_3_^-^ (mg L-1) | P-PO_4_^3-^ (mg L^-1^) |
| --- | --- | --- | --- | --- | --- | --- | --- | --- | --- | --- | --- | --- |
| C1 | Spring | 21.30 | 8.65 | 0.45 | 350.00 | 7.98 | 1.10 | 100.00 | 391.60 | 0.0048 | 5.1800 | 0.2320 |
| C1 | Summer | 26.60 | 7.78 | 0.55 | 323.00 | 8.34 | 3.30 | 70.00 | 176.22 | 0.7576 | 0.1706 | 0.0174 |
| C1 | Autumn | 17.40 | 9.06 | 0.60 | 315.00 | 8.42 | 2.20 | 155.00 | 418.30 | 0.0512 | 0.3294 | 0.0264 |
| C1 | Winter | 9.50 | 11.22 | 0.79 | 378.00 | 8.71 | 1.18 | 160.00 | 320.40 | 0.0736 | 0.8059 | 0.0253 |
| C2 | Spring | 21.60 | 8.63 | 0.63 | 347.00 | 8.01 | 1.33 | 130.00 | 409.40 | 0.0056 | 5.2800 | 0.2380 |
| C2 | Summer | 26.80 | 7.73 | 0.53 | 317.00 | 8.20 | 2.67 | 75.00 | 178.00 | 0.5600 | 0.1765 | 0.0124 |
| C2 | Autumn | 17.30 | 9.17 | 0.42 | 310.00 | 8.42 | 2.12 | 125.00 | 311.50 | 0.0504 | 0.3471 | 0.0253 |
| C2 | Winter | 9.70 | 11.13 | 0.75 | 385.00 | 8.77 | 2.20 | 145.00 | 338.20 | 0.0728 | 0.6000 | 0.0135 |
| N1 | Spring | 21.30 | 8.49 | 0.62 | 345.00 | 7.69 | 0.94 | 135.00 | 356.00 | 0.0056 | 5.2118 | 0.2275 |
| N1* | Summer |  |  |  |  |  |  |  |  |  |  |  |
| N1 | Autumn | 17.70 | 9.07 | 0.64 | 297.00 | 8.27 | 1.73 | 275.00 | 510.86 | 0.0504 | 0.4471 | 0.0230 |
| N1 | Winter | 9.80 | 11.09 | 0.97 | 376.00 | 8.90 | 1.81 | 125.00 | 348.88 | 0.0688 | 0.5941 | 0.0180 |
| N2 | Spring | 22.90 | 7.90 | 0.39 | 337.00 | 7.64 | 1.02 | 120.00 | 391.60 | 0.0088 | 5.2176 | 0.2382 |
| N2 | Summer | 26.40 | 7.56 | 0.29 | 324.00 | 8.00 | 2.59 | 70.00 | 105.02 | 0.0232 | 0.2059 | 0.0146 |
| N2 | Autumn | 19.80 | 8.34 | 0.24 | 265.00 | 8.31 | 2.51 | 175.00 | 224.28 | 0.1424 | 1.2529 | 0.0039 |
| N2 | Winter | 9.80 | 11.06 | 0.63 | 374.00 | 8.94 | 1.18 | 125.00 | 320.40 | 0.0680 | 0.8529 | 0.0163 |
| N3 | Spring | 21.10 | 8.68 | 0.73 | 338.00 | 7.70 | 1.41 | 110.00 | 391.60 | 0.0072 | 5.2353 | 0.2416 |
| N3 | Summer | 26.00 | 7.76 | 1.02 | 305.00 | 8.04 | 2.51 | 70.00 | 133.50 | 0.0408 | 0.1412 | 0.0202 |
| N3 | Autumn | 20.00 | 8.86 | 0.66 | 289.00 | 8.32 | 0.94 | 150.00 | 222.50 | 0.0912 | 0.5118 | 0.0028 |
| N3 | Winter | 9.70 | 11.04 | 1.35 | 375.00 | 8.91 | 1.10 | 155.00 | 339.98 | 0.0688 | 0.5765 | 0.0135 |
| N4 | Spring | 20.90 | 8.72 | 0.82 | 347.00 | 7.74 | 1.10 | 100.00 | 391.60 | 0.0048 | 5.2300 | 0.2370 |
| N4 | Summer | 25.80 | 7.88 | 0.91 | 305.00 | 8.04 | 2.51 | 70.00 | 124.60 | 0.0344 | 0.2176 | 0.0174 |
| N4 | Autumn | 17.70 | 9.42 | 0.45 | 245.00 | 8.36 | 1.49 | 150.00 | 236.74 | 0.0416 | 0.7059 | 0.0062 |
| N4 | Winter | 9.40 | 11.20 | 1.23 | 373.00 | 8.90 | 1.26 | 150.00 | 338.20 | 0.0664 | 0.6000 | 0.0180 |
| N5 | Spring | 21.40 | 8,65 | 0.63 | 348.00 | 7.83 | 1.33 | 105.00 | 338.20 | 0.0024 | 5.2600 | 0.2320 |
| N5 | Summer | 26.40 | 7.78 | 0.23 | 293.00 | 8.15 | 2.83 | 70.00 | 158.42 | 0.0368 | 0.1824 | 0.0146 |
| N5 | Autumn | 17.50 | 9.10 | 0.28 | 292.00 | 8.38 | 2.36 | 75.00 | 421.86 | 0.0504 | 0.4529 | 0.0208 |
| N5 | Winter | 9.70 | 11.12 | 0.84 | 379.00 | 8.82 | 1.18 | 170.00 | 309.72 | 0.0520 | 0.5118 | 0.0152 |

*N1 Summer - dry
