# Supplementary material for: Taxonomic and Functional Metrics of Ciliates and Amoeboid Protists in Response to Stream Revitalization
Source: Front Microbiol. 2022 Apr 1;13:842395. doi: 10.3389/fmicb.2022.842395 (PMC9010972; doi:10.3389/fmicb.2022.842395)
Supplement: Supplementary file 4 [file Table_4.DOCX]

**Supplementary table S4.** GLMM (full model) output showing main effects of environmental factors (fixed effects) on taxonomic and functional metrics of ciliate assemblages, with season and replicate as random effects. Statistically significant effects (p<0.05) are reported in bold. Legend: F – F statistic; d.f. – degrees of freedom.

| **One-month** | **Assemblage parameter** | **Environmental parameter** | **F** | **p** | **d.f.** | **d.f corrected** | **Coefficient** |
| --- | --- | --- | --- | --- | --- | --- | --- |
|  | Abundance | COD | 0.137 | 0.713 | 1 | 50 | -0.149 |
|  |  | Nitrites | 3.079 | 0.085 | 1 | 50 | 3.947 |
|  |  | TWH | 2.071 | 0.156 | 1 | 50 | 0.004 |
|  | Species richness | COD | 1.117 | 0.296 | 1 | 50 | 0.234 |
|  |  | Nitrites | 0.013 | 0.910 | 1 | 50 | -0.089 |
|  |  | TWH | 0.178 | 0.675 | 1 | 50 | 0.001 |
|  | True diversity (Shannon) | COD | 0.469 | 0.497 | 1 | 50 | 0.068 |
|  |  | Nitrites | 2.542 | 0.117 | 1 | 50 | -0.592 |
|  |  | TWH | 2.217 | 0.143 | 1 | 50 | 0.001 |
|  | True diversity (Simpson) | COD | 0.141 | 0.709 | 1 | 50 | -0.063 |
|  |  | Nitrites | 0.952 | 0.334 | 1 | 50 | -0.608 |
|  |  | TWH | 0.717 | 0.334 | 1 | 50 | 0.001 |
|  | FDis | COD | 1.166 | 0.285 | 1 | 50 | -0.430 |
|  |  | Nitrites | 0.064 | 0.801 | 1 | 50 | 0.429 |
|  |  | TWH | 7.714 | **0.008** | 1 | 50 | 0.007 |
|  | RaoQ | COD | 0.732 | 0.396 | 1 | 50 | -0.511 |
|  |  | Nitrites | 4.497 | **0.039** | 1 | 50 | -4.838 |
|  |  | TWH | 3.742 | 0.059 | 1 | 50 | 0.007 |
| **Two-months** | Abundance | DO | 1.162 | 0.312 | 1 | 43 | 0.476 |
|  |  | Conductivity | 4.059 | 0.050 | 1 | 43 | -0.010 |
|  |  | pH | 0.719 | 0.395 | 1 | 43 | 1.604 |
|  |  | COD | 5.844 | **0.009** | 1 | 43 | -0.776 |
|  |  | Alkalinity | 1.763 | 0.187 | 1 | 43 | -0.006 |
|  |  | TWH | 0.914 | 0.488 | 1 | 43 | 0.003 |
|  |  | Ortho | 0.971 | 0.348 | 1 | 43 | 5.925 |
|  | Species richness | DO | 0.484 | 0.490 | 1 | 43 | 0.126 |
|  |  | Conductivity | 8.348 | **0.006** | 1 | 43 | -0.010 |
|  |  | pH | 3.594 | 0.065 | 1 | 43 | 1.276 |
|  |  | COD | 0.547 | 0.464 | 1 | 43 | 0.173 |
|  |  | Alkalinity | 1.672 | 0.203 | 1 | 43 | -0.004 |
|  |  | TWH | 0.223 | 0.639 | 1 | 43 | 0.001 |
|  |  | Ortho | 1.223 | 0.275 | 1 | 43 | 3.340 |
|  | True diversity (Shannon) | DO | 0.284 | 0.597 | 1 | 43 | -0.085 |
|  |  | Conductivity | 8.976 | **0.005** | 1 | 43 | -0.009 |
|  |  | pH | 9.489 | **0.004** | 1 | 43 | 1.929 |
|  |  | COD | 1.811 | 0.185 | 1 | 43 | 0.264 |
|  |  | Alkalinity | 0.186 | 0.668 | 1 | 43 | 0.001 |
|  |  | TWH | 0.063 | 0.803 | 1 | 43 | 0.000 |
|  |  | Ortho | 4.022 | **0.051** | 1 | 43 | 5.522 |
|  | True diversity (Simpson) | DO | 0.746 | 0.393 | 1 | 43 | -0.129 |
|  |  | Conductivity | 9.639 | **0.003** | 1 | 43 | -0.009 |
|  |  | pH | 12.055 | **0.001** | 1 | 43 | 2.021 |
|  |  | COD | 1.714 | 0.197 | 1 | 43 | 0.234 |
|  |  | Alkalinity | 0.239 | 0.628 | 1 | 43 | -0.001 |
|  |  | TWH | 0.080 | 0.779 | 1 | 43 | 0.000 |
|  |  | Ortho | 4.810 | **0.034** | 1 | 43 | 5.685 |
|  | FDis | DO | 1.095 | 0.436 | 1 | 43 | 0.628 |
|  |  | Conductivity | 0.659 | 0.465 | 1 | 43 | -0.019 |
|  |  | pH | 2.076 | 0.071 | 1 | 43 | 2.954 |
|  |  | COD | 1.462 | 0.233 | 1 | 43 | 1.693 |
|  |  | Alkalinity | 2.035 | 0.137 | 1 | 43 | 0.010 |
|  |  | TWH | 0.172 | 0.715 | 1 | 43 | -0.002 |
|  |  | Ortho | 1.252 | 0.273 | 1 | 43 | 13.017 |
|  | RaoQ | DO | 0.761 | 0.133 | 1 | 43 | 0.635 |
|  |  | Conductivity | 2.293 | 0.263 | 1 | 43 | -0.012 |
|  |  | pH | 3.193 | 0.071 | 1 | 43 | 4.823 |
|  |  | COD | 7.844 | **0.009** | 1 | 43 | 3.018 |
|  |  | Alkalinity | 0.569 | 0.425 | 1 | 43 | 0.004 |
|  |  | TWH | 0.170 | 0.717 | 1 | 43 | -0.003 |
|  |  | Ortho | 1.496 | 0.107 | 1 | 43 | 6.979 |
