# Supplementary material for: Taxonomic and Functional Metrics of Ciliates and Amoeboid Protists in Response to Stream Revitalization
Source: Front Microbiol. 2022 Apr 1;13:842395. doi: 10.3389/fmicb.2022.842395 (PMC9010972; doi:10.3389/fmicb.2022.842395)
Supplement: Supplementary file 5 [file Table_5.DOCX]

**Supplementary table S5**. GLMM (full model) output showing main effects of environmental factors (fixed effects) on taxonomic and functional metrics of amoeboid protist assemblages, with season and replicate as random effects. Statistically significant effects (p<0.05) are reported in bold. Legend: F – F statistic; d.f. – degrees of freedom.

| **One-month** | **Assemblage parameter** | **Environmental parameter** | **F** | **p** | **d.f.** |  | **Coefficient** |
| --- | --- | --- | --- | --- | --- | --- | --- |
|  | Abundance | TWH | 3.330 | 0.074 | 1 | 50 | -0.004 |
|  |  | Nitrites | 2.463 | 0.123 | 1 | 50 | 2.164 |
|  |  | COD | 4.834 | **0.033** | 1 | 50 | 0.690 |
|  | Species richness | TWH | 2.685 | 0.108 | 1 | 50 | 0.002 |
|  |  | Nitrites | 8.021 | **0.007** | 1 | 50 | -3.526 |
|  |  | COD | 1.629 | 0.208 | 1 | 50 | 0.169 |
|  | True diversity (Shannon) | TWH | 3.283 | 0.076 | 1 | 50 | 0.001 |
|  |  | Nitrites | 6.716 | **0.012** | 1 | 50 | -1.404 |
|  |  | COD | 0.379 | 0.541 | 1 | 50 | 0.050 |
|  | True diversity (Simpson) | TWH | 3.888 | 0.054 | 1 | 50 | 0.002 |
|  |  | Nitrites | 5.413 | **0.024** | 1 | 50 | -2.208 |
|  |  | COD | 0.655 | 0.422 | 1 | 50 | 0.100 |
|  | FDis | TWH | 3.475 | 0.068 | 1 | 50 | -0.011 |
|  |  | Nitrites | 3.805 | 0.057 | 1 | 50 | -17.316 |
|  |  | COD | 0.146 | 0.704 | 1 | 50 | -0.318 |
|  | RaoQ | TWH | 2.742 | 0.104 | 1 | 50 | -0.008 |
|  |  | Nitrites | 4.466 | **0.040** | 1 | 50 | -15.668 |
|  |  | COD | 0.370 | 0.546 | 1 | 50 | -0.437 |
| **Two-months** | Abundance | DO | 14.348 | **<0.001** | 1 | 43 | -1.211 |
|  |  | Conductivity | 5.605 | **0.022** | 1 | 43 | 0.013 |
|  |  | pH | 5.347 | **0.026** | 1 | 43 | 2.548 |
|  |  | COD | 3.861 | 0.056 | 1 | 43 | -0.611 |
|  |  | Alkalinity | 6.992 | **0.011** | 1 | 43 | -0.013 |
|  |  | TWH | 17.087 | **<0.001** | 1 | 43 | 0.009 |
|  |  | Ortho | 1.893 | 0.176 | 1 | 43 | -6.440 |
|  | Species richness | DO | 29.918 | **<0.001** | 1 | 43 | -1.257 |
|  |  | Conductivity | 13.119 | **<0.001** | 1 | 43 | 0.022 |
|  |  | pH | 1.949 | 0.170 | 1 | 43 | 0.988 |
|  |  | COD | 0.149 | 0.701 | 1 | 43 | -0.091 |
|  |  | Alkalinity | 0.141 | 0.709 | 1 | 43 | 0.002 |
|  |  | TWH | 17.196 | **<0.001** | 1 | 43 | 0.008 |
|  |  | Ortho | 5.915 | **0.019** | 1 | 43 | -8.005 |
|  | True diversity (Shannon) | DO | 26.864 | **<0.001** | 1 | 43 | -1.095 |
|  |  | Conductivity | 12.899 | **<0.001** | 1 | 43 | 0.016 |
|  |  | pH | 1.774 | 0.190 | 1 | 43 | 0.891 |
|  |  | COD | 0.598 | 0.444 | 1 | 43 | -0.174 |
|  |  | Alkalinity | 0.661 | 0.421 | 1 | 43 | 0.003 |
|  |  | TWH | 12.780 | **<0.001** | 1 | 43 | 0.006 |
|  |  | Ortho | 5.127 | **0.029** | 1 | 43 | -6.502 |
|  | True diversity (Simpson) | DO | 26.470 | **<0.001** | 1 | 43 | -1.028 |
|  |  | Conductivity | 13.285 | **<0.001** | 1 | 43 | 0.016 |
|  |  | pH | 1.276 | 0.265 | 1 | 43 | 0.707 |
|  |  | COD | 1.051 | 0.311 | 1 | 43 | -0.217 |
|  |  | Alkalinity | 0.465 | 0.499 | 1 | 43 | 0.003 |
|  |  | TWH | 11.517 | **0.001** | 1 | 43 | 0.006 |
|  |  | Ortho | 5.754 | **0.021** | 1 | 43 | -6.580 |
|  | FDis | DO | 4.951 | **0.031** | 1 | 43 | -1.572 |
|  |  | Conductivity | 0.523 | 0.473 | 1 | 43 | -0.009 |
|  |  | pH | 11.541 | **0.001** | 1 | 43 | 7.378 |
|  |  | COD | 4.779 | **0.034** | 1 | 43 | -1.534 |
|  |  | Alkalinity | 1.424 | 0.239 | 1 | 43 | 0.014 |
|  |  | TWH | 13.428 | **<0.001** | 1 | 43 | -0.018 |
|  |  | Ortho | 6.533 | **0.014** | 1 | 43 | 23.133 |
|  | RaoQ | DO | 0.953 | 0.334 | 1 | 43 | -0.727 |
|  |  | Conductivity | 0.042 | 0.839 | 1 | 43 | -0.003 |
|  |  | pH | 3.950 | 0.053 | 1 | 43 | 4.859 |
|  |  | COD | 0.522 | 0.474 | 1 | 43 | -0.526 |
|  |  | Alkalinity | 0.017 | 0.896 | 1 | 43 | 0.002 |
|  |  | TWH | 5.567 | **0.023** | 1 | 43 | -0.012 |
|  |  | Ortho | 3.839 | 0.057 | 1 | 43 | 19.350 |
